# Multiplex quantitative analysis of microRNA expression via exponential isothermal amplification and conformation-sensitive DNA separation

**DOI:** 10.1038/s41598-017-11895-6

**Published:** 2017-09-12

**Authors:** Jeongkyeong Na, Gi Won Shin, Heehwa G. Son, Seung-Jae V. Lee, Gyoo Yeol Jung

**Affiliations:** 1 Department of Chemical Engineering, Pohang University Science and Technology, Pohang, Gyeongbuk 790-784 Korea; 2 Institute of Environmental and Energy Technology, Pohang University of Sciences and Technology, Pohang, Gyeongbuk 790-784 Korea; 3 Department of Life Sciences, Pohang University of Sciences and Technology, Pohang, Gyeongbuk 790-784 Korea; 4 Information Technology Convergence Engineering, Pohang University of Science and Technology, Pohang, Gyeongbuk 790-784 Korea; 5 School of Interdisciplinary Bioscience and Bioengineering, Pohang University of Sciences and Technology, Pohang, Gyeongbuk 790-784 Korea; 6 Present Address: Division of Oncology, Department of Medicine, Stanford University School of Medicine, Stanford, California 94305 USA

## Abstract

Expression profiling of multiple microRNAs (miRNAs) generally provides valuable information for understanding various biological processes. Thus, it is necessary to develop a sensitive and accurate miRNA assay suitable for multiplexing. Isothermal exponential amplification reaction (EXPAR) has received significant interest as an miRNA analysis method because of high amplification efficiency. However, EXPAR cannot be used for a broader range of applications owing to limitations such as complexity of probe design and lack of proper detection method for multiplex analysis. Here, we developed a sensitive and accurate multiplex miRNA profiling method using modified isothermal EXPAR combined with high-resolution capillary electrophoresis-based single-strand conformation polymorphism (CE-SSCP). To increase target miRNA specificity, a stem-loop probe was introduced instead of a linear probe in isothermal EXPAR to allow specific amplification of multiple miRNAs with minimal background signals. CE-SSCP, a conformation-dependent separation method, was used for detection. Since CE-SSCP eliminates the need for probes to have different lengths, easier designing of probes with uniform amplification efficiency was possible. Eight small RNAs comprising six miRNAs involved in *Caenorhabditis elegans* development and two controls were analyzed. The expression patterns obtained using our method were concordant with those reported in previous studies, thereby supporting the proposed method’s robustness and utility.

## Introduction

MicroRNAs (miRNAs) are small non-coding RNAs that range from 18 to 25 bp in length and are found in a broad range of plants and mammals. miRNAs are known to regulate gene expression via translational repression or target degradation by hybridizing with target mRNA. miRNAs are critical regulators involved in various biological processes, such as development, differentiation, metabolism, and apoptosis. Thus, in addition to mRNAs, miRNA expression patterns should be measured to obtain a detailed understanding of biological processes^1, 2^. In addition, changes in miRNA expression levels are commonly observed in several diseases, such as cancers, cardiovascular diseases, rheumatic diseases, and neurological disorders^3–5^. In each case, aberrant expression is observed in a group of miRNAs rather than a single miRNA^4, 6–8^. As a result, researchers have increasingly employed multiplex analysis of miRNA expression not only to investigate the biological processes regulated by miRNAs, but also to identify potential diagnostic markers for use in the clinical setting.

miRNA expression profiling is challenging owing to the intrinsic properties of miRNAs, such as short lengths and high sequence homology among family members. Various methods have been used to investigate miRNA expression patterns, including northern blotting, quantitative RT-PCR (qRT-PCR), rolling circle amplification, loop-mediated isothermal amplification (LAMP), and isothermal exponential amplification reaction (EXPAR)^9–13^. However, none of these methods are capable of analyzing multiple miRNAs in a single assay. Northern blotting^14^ and qRT-PCR^15^ are currently regarded as the standard methods for analyzing miRNA expression. However, Northern blotting is not sensitive enough to detect less abundant miRNAs, which require large amounts of starting material (10–30 μg of total RNA) for analysis, and other miRNA assays require total RNA amounts in the nanogram range^2, 16, 17^. Moreover, Northern blotting is labor-intensive and time-consuming. qRT-PCR allows sensitive and accurate quantification of miRNA but has been successfully used in only a few studies using singleplex assays. Furthermore, the short lengths of miRNAs makes PCR primer design complicated^18^, and limited availability of fluorescence dyes allows multiplexing of only up to five targets^19^. EXPAR^9–12^, the most recent miRNA detection method, combines strand extension and single-strand nicking to rapidly amplify miRNA at high amplification efficiency, reaching up to 10^6^- to 10^9^-fold amplification within a few minutes under isothermal conditions. However, isothermal EXPAR also has limited applicability for multiplex miRNA assays because real-time monitoring of amplification is used for detection as in qRT-PCR methods.

On the other hand, ligation-mediated PCR assays can simultaneously measure multiple short-length miRNAs with higher specificity and can discriminate between miRNA species bearing only single nucleotide differences^6, 20^. Using capillary electrophoresis (CE) as the detection method, ligation-mediated PCR can be used to analyze up to eight miRNA species in a single reaction^20^. However, considering that CE separates multiple miRNAs based on differing lengths, this method requires the probes to have DNA sequence tags with varying lengths, which can lead to bias in quantitative analysis.

In the present study, we developed a multiplex miRNA quantification assay that combines a modified isothermal EXPAR method and high-resolution capillary electrophoresis single strand conformation polymorphism (CE-SSCP) analysis. We designed an assay based on six miRNAs known to be involved in *Caenorhabditis elegans* developmental processes, as well as both internal and external controls. Small RNA extracts were obtained from two different developmental stages and quantitatively analyzed and compared with previously reported qRT-PCR results^8^. Our assay utilizes stem-loop RT primers, which exhibit 100 times higher specificity compared to linear primers^15^. CE-SSCP was used to detect the amplified probes; this method is suitable for analyzing multiple probes using a single fluorescent tag. Unlike conventional CE, which requires the probes to have different lengths^21, 22^, CE-SSCP can separate DNA molecules based on conformational differences arising from sequence diversity. In particular, using Pluronic polymer as the separation matrix will dramatically increase the resolution, so that up to 20 targets can be simultaneously analyzed in a single assay^21–25^. Eight small RNA species demonstrate the strong potential of our multiplex assay in miRNA applications.

## Results and Discussion

### Overview of miRNA assay

**Figure 1:**
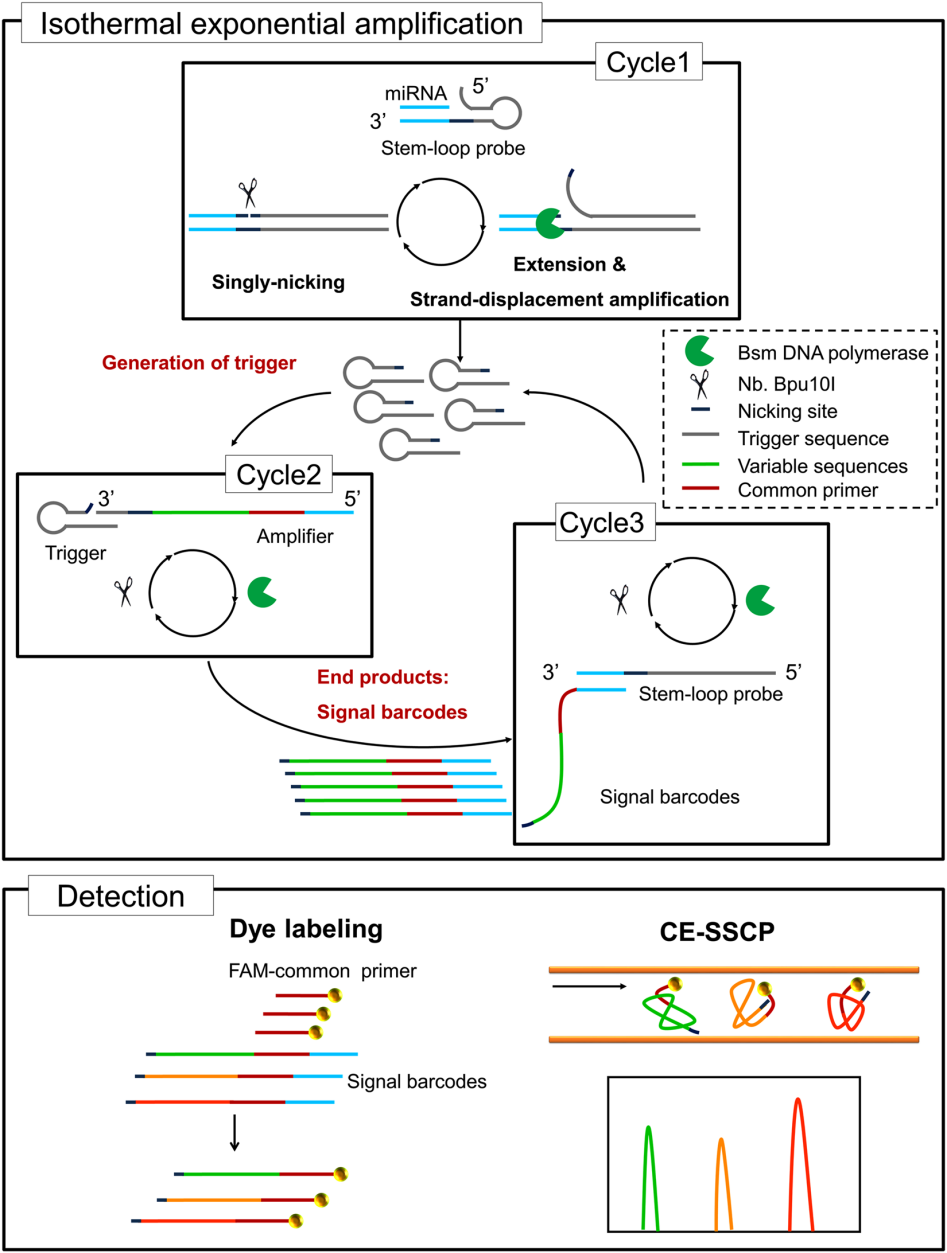
Schematic illustration of isothermal exponential amplification (EXPAR) and CE-SSCP analysis. Exponential amplification of miRNA signals under isothermal conditions through Cycles 1, 2, and 3. **Cycle 1:** Generation of triggers from miRNAs by using stem-loop probe. When miRNAs hybridize with stem-loop structured probe, triggers are generated through repetitive extension, single-strand nicking, and strand-displacement by Bsm DNA polymerase and Nb.Bpu10I. **Cycle 2:** Amplification of signal barcodes from triggers generated in Cycle 1. When the trigger hybridizes with the amplifier, signal barcodes are generated in the same way as triggers are generated (i.e. repetitive extension, single-strand nicking, and strand displacement). **Cycle 3:** Further generation of triggers for exponential amplification of signal barcodes. Triggers can be generated from both signal barcode and stem-loop probes. Signal barcodes are detected by dye labeling and CE-SSCP. For CE-SSCP analysis, the signal barcode must be labeled with a fluorescent dye. Products can be analyzed simultaneously via CE-SSCP using fluorescently labeled common primer in the subsequent linear amplification step.

The miRNA assay developed in this study consists of three cycles and is designed to exponentially amplify signals specific to the target miRNA species (Fig. 1 and Supplementary Figure S1). In Cycle 1, multiple trigger molecules are generated from single miRNA molecules by repeated strand extension and nicking steps. Strand displacement activity of the DNA polymerase enables initial extension of the hybridized stem-loop probe and additional extension after nicking and releasing of triggers. Stem-loop probes were designed to contain a nicking enzyme recognition sequence between the trigger sequence at the 5′ end and an anti-miRNA sequence at the 3′ end (Supplementary Figure S1A and Supplementary Table S2A). The previous study by Chen *et al*. demonstrated that using stem-loop RT primers results in 100 times higher efficiency than the linear RT primer^15^. The stem-loop structure provides many advantages for miRNA assay. First, base stacking interactions that form the RNA-DNA heteroduplex enhance thermal stability, which could increase reverse transcription efficiency in case of relatively short RNAs. Second, the spatially constrained stem-loop structure can improve the specificity of primer only to 3′ end of mature small RNA, which prevents binding to primary miRNA (pri-miRNA) and precursor miRNA (pre-miRNA)^26, 27^. Third, the stem-loop structure could minimize non-specific interactions with genomic DNA. The primer cannot bind to untranscribed miRNA sequences in long genomic DNA fragments; thus, the protocol does not require prior DNase treatment. In Cycle 2, the signal barcodes are amplified by repeated nicking and extension of the amplifiers (Supplementary Figure S1A and Supplementary Table S2B). Cycle 2 reactions can only be initiated when the amplifier is hybridized with the triggers obtained in Cycle 1. The signal barcodes contain labeling primer sequences and a region with variable sequence. The latter will be used to separate assay products from each miRNA species in the final CE-SSCP analysis. Cycle 3 is designed to generate additional triggers via further extension of signal barcodes generated in Cycle 2. The last 15 bases of the signal barcodes can hybridize to the stem-loop probes, by which the additional extension and nicking steps are initiated^28^.

The signal barcodes generated by Cycle 2 during the exponential amplification are subsequently labeled with fluorescence and analyzed by CE-SSCP (Fig. 1). The signal barcodes are variable sequences which are designed to have various single-strand DNA (ssDNA) folding stability. In CE-SSCP analysis, similar-sized ssDNA molecules migrate in different rates according to their folding stability. The stability can be described as free energy difference (ΔG) between denatured and folded states. Although prediction of tertiary folding structure is not currently available, ΔG of secondary folding also shows a good correlation with CE-SSCP mobility^28^. We used MFOLD software (http://unafold.rna.albany.edu/?q=DINAMelt/Two-state-folding) to predict secondary structure of candidate sequences that range from 30 to 40 bp in length.

The probes used in this study were designed for seven target miRNAs and snoRNA U18, whose expression patterns have been previously reported^8^. The six target miRNAs included three miRNAs (lin-4, mir-48, and mir-84) whose expression levels are known to vary depending on the developmental timing and three constantly expressed tissue-specific miRNAs in *C*. *elegans*. Two types of reference probes were used for normalization. One of the controls was an endogenous control (snoRNA U18) that exhibits relatively stable expression^29^, and the other was the spike-in control mir-159a from *Arabidopsis* ^30^. The spike-in control prevents false positive signal amplification when the other target miRNAs are all absent.

### Validation of cleavage and extension reactions

**Figure 2:**
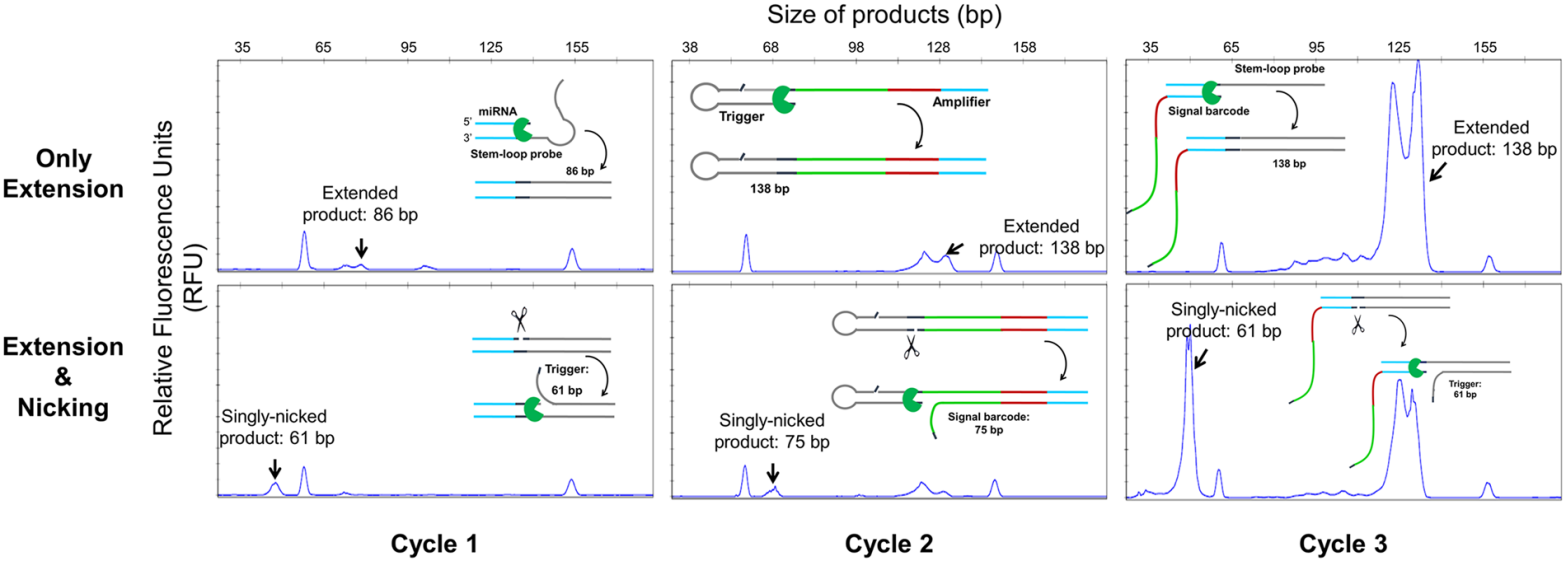
Confirmation of extension and cleavage reactions in Cycles 1, 2, and 3 in isothermal EXPAR. Product sizes of extended and singly nicked products for mir-48 in each cycle were analyzed via denaturing CE, in which FAM-dCTP was added to label the reaction products in the test reactions. The expected size of the product is indicated by a black arrow in each electropherogram.

In designing the miRNA assay, the extension and cleavage reactions in every cycle were validated individually. Each cycle must be properly carried out to achieve exponential amplification. For validation, the extension and cleavage reaction products were separated via denaturing CE. CE resolves DNA molecules based on size under denaturing conditions. For all extension reactions, two single-stranded nucleic acids were synthesized, one of which was used as primer, while the other served as the template for polymerase extension. For example, miRNA and stem-loop probe were used as the primer and template for the extension reaction in Cycle 1, respectively. The primers and templates used in Cycles 2 and 3 were the intermediate products obtained from other cycles in the actual assay. However, we used individually synthesized primers and templates to allow independent evaluation for each cycle. In addition, subsequent nicking enzyme reactions were also tested using a fraction of the extended products from all the cycles. As shown in Fig. 2 and Supplementary Figure S2, all extended and nicked products were detected at their expected sizes.

### Optimization of miRNA assay conditions

To maximize the signal amplification efficiency of our miRNA assay, we tested various concentrations of enzymes and probes, as well as reaction period. Appropriate combination of the Bsm DNA polymerase and Nb.Bpu10I nicking enzyme is important to achieve optimal efficiency^12, 31^. Therefore, we tested different quantities of DNA polymerase and nicking enzymes. The nicking enzyme was tested in the range of 4–8 U, while with the DNA polymerase was tested at 0.5 and 1 U (Supplementary Figure S3). Among the tested combinations, 0.5 U of Bsm DNA polymerase combined with 7 U of Nb.Bpu10I nicking enzyme produced the maximum signal intensity. Interestingly, a significant decrease in signal intensity was observed when more than 7 U of the nicking enzyme was used, which is likely caused by non-specific endonuclease activity at higher concentrations of the nicking enzyme.

**Figure 3:**
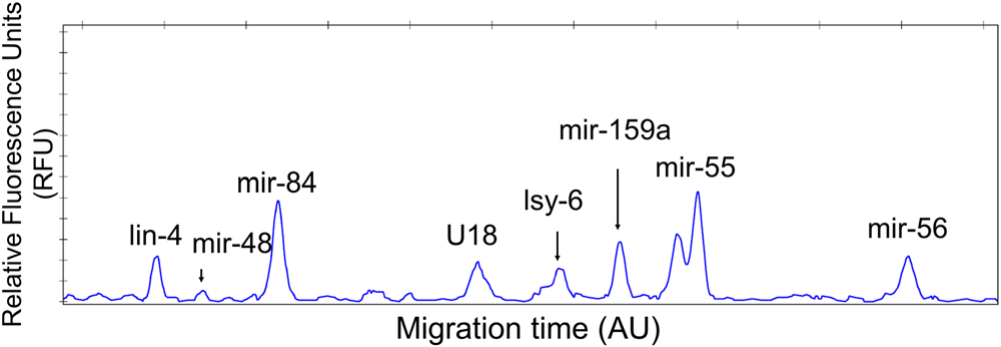
Electropherogram for multiplex detection of miRNAs from *Caenorhabditis elegans* total RNA. Development-associated miRNAs in *C*. *elegans* (lin-4, mir-48, mir-84, lsy-6, mir-55, and mir-56) and two controls (snoRNA U18 and mir-159a) were detected via CE-SSCP analysis after the dye-labeling reaction.

Subsequently, the concentrations of stem-loop probes and amplifiers were optimized, and the optimal reaction time was determined after conducting a series of experiments. Various ratios between stem-loop probes and amplifiers (1:1, 1:2, 1:3, 1:4, 2:1, 3:1, and 4:1) were tested while fixing the smaller amount to 30 fmol. Reaction times ranging from 30 min to 4 h were tested to obtain saturated amplification signals. Maximum signal intensity could be obtained when the optimal concentrations of stem-loop probe (60 fmol) and amplifier (30 fmol) were used with a 3-h reaction period. Using the optimized conditions, the detection limit was determined to be approximately 2 fmol, which is comparable to those of other isothermal EXPAR methods^11, 32^. In addition, the currently described miRNA assay can be used to simultaneously analyze up to eight small RNAs, including two references, under optimal reaction conditions (Fig. 3). The validity of the multiplex assay was confirmed by analyzing single-species miRNA samples (Supplementary Figure S4). The assay generated only the peaks corresponding to the miRNA species included in the samples, and the intensity of the peaks changed accordingly with the input miRNA amount.

During the developmental process of *C*. *elegans*, up to tenfold changes in miRNA expression levels are frequently observed^33–35^. Therefore, we evaluated the quantification capability of our miRNA assay within a biologically relevant dynamic range. Various mixtures were prepared using seven synthetic miRNAs, in which the variation in miRNA expression levels were demonstrated. A strong correlation was observed between the measured and expected miRNA fold change values (Supplementary Figure S5), and measurements reproducibly showed 5% average standard deviation within triplicate experiments.

### Analysis of dynamic changes in miRNA expression levels

**Figure 4:**
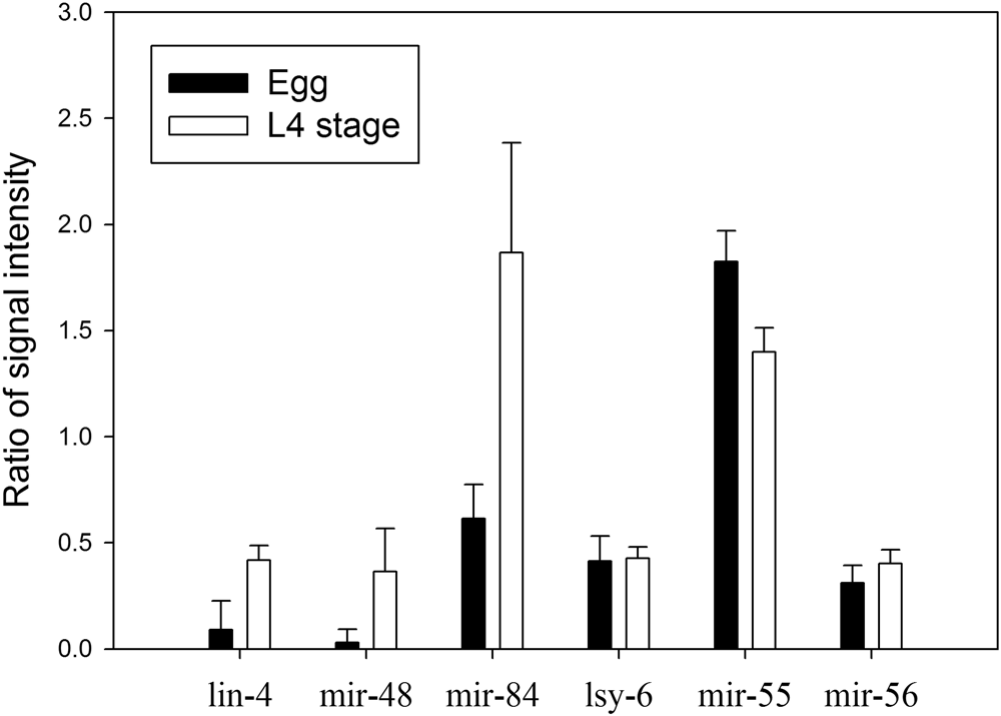
Expression profiling of six miRNAs in *C*. *elegans* via the modified isothermal EXPAR combined with CE-SSCP analysis. Expression profiles were compared between the egg and L4 stages. lsy-6, mir-55, and mir-56 showed no significant changes in their expression levels, whereas lin-4, mir-48, and mir-84 were upregulated. Three independent samples were prepared for each stage, and all measurements were performed in triplicate. The mean and standard deviation of the nine values were plotted.

Our miRNA profiling method was validated using a set of *C*. *elegans* miRNAs that have been previously reported to be differentially expressed between the egg and fourth larval (L4) stages^8, 36^. Three independently grown pairs of worms were prepared, and the average and standard deviation of the miRNA fold change values were compared between the two stages (Fig. 4). Similar expression levels for lsy-6, mir-55, and mir-56 were observed between the two stages; on the other hand, lin-4, mir-48, and mir-84 were upregulated in the L4 stage. In five out of the six target miRNAs analyzed, the fold change values determined using the currently described miRNA assay were concordant with the those previously reported by Karp *et al*.^34^. However, lsy-6, which was detected using our method in both larval stages, was not detected by Karp *et al*. Although qPCR is considered the gold standard method for miRNA quantitation, it is inefficient for multiplex analysis and suffers from inherent PCR bias^37–39^. The results showed that our method, which is based on isothermal EXPAR and CE-SSCP, enables highly sensitive multiplex miRNA analysis compared to qPCR-based methods.

## Conclusions

We demonstrate a modified isothermal EXPAR method combined with CE-SSCP that can be used to quantify the expression of multiple miRNAs with high specificity using stem-loop probes. Furthermore, multiplexing power was increased to an eight-plex assay by using high-resolution CE-SSCP as the detection method. Conventional isothermal EXPAR methods are generally able to simultaneously measure the expression levels of only one to three miRNAs^9–11, 13, 40^. Furthermore, combining isothermal EXPAR with CE-SSCP presents the advantage of having universal amplifiers that can amplify different sets of miRNA targets. Once the target signal is amplified, isothermal EXPAR can be used to amplify products that do not include target miRNA sequence. The performance of the currently described isothermal EXPAR combined with CE-SSCP was validated by measuring the fold changes of six miRNAs in both synthesized miRNA mixtures and in total RNA extract of *C*. *elegans*. Introducing the high-resolution CE-SSCP system allows the expression analysis of multiple miRNAs without length-dependent bias and thus offers great potential for precise expression profiling of multiple miRNAs involved in various biological processes, including cancer progression.

## Materials and Methods

### RNA preparation

miRNAs were obtained either by purchasing commercially synthesized RNA (Bioneer, Daejeon, Korea) or via *in vitro* transcription (Supplementary Table S1). *In vitro* transcription was performed using a double-stranded DNA template with the following sequence: 5′-GAATT*CTAATACGACTCACTATA*CCC-(miRNA sequence)-3′ (italicized letters indicate T7 RNA polymerase promoter sequence). Reactions contained 1 μM DNA template, 7.5 mM dNTP, 10 U/μL T7 RNA polymerase, 1 U/μL RiboLock RNase inhibitor, and 1 × T7 RNA polymerase buffer [40 mM Tris HCl (pH 7.9), 10 mM NaCl, 10 mM dithiothreitol, 6 mM MgCl_2_, and 2 mM spermidine] and were incubated at 37 °C overnight. Synthesized miRNAs were purified via PAGE followed by ethanol precipitation. T7 RNA polymerase and RiboLock inhibitor used for *in vitro* transcription were purchased from Thermo Fisher Scientific Inc. (Waltham, MA, USA).

Total RNA was isolated as previously described with minor modifications^41^. Wild-type *C*. *elegans* strain N2 worms in two developmental stages were collected. While maintaining the temperature at 20 °C, embryos were first obtained by bleaching gravid adults. Fourth larval (L4) stage worms were then harvested after culturing the bleached eggs. Total RNA was extracted from three independently prepared sample pairs using RNAiso plus kit (Takara, Shiga, Japan).

### Validation of cleavage and extension in each cycle

To verify the extension and cleavage reactions for each cycle, generated products in each reaction were labeled using FAM-dCTP (Biorbyt, Cambridge, UK) and analyzed via denaturing CE separation. Extension reactions were performed in 20-µL reaction volumes containing 40 pmol template, 40 pmol probe, Bsm DNA polymerase (8 U), 60 pmol FAM-dCTP, 250 μM dNTP, and 1× Buffer R [10 mM Tris-HCl (pH 8.5), 100 mM KCl, 10 mM MgCl_2_, and 0.1 mg/mL BSA]. Reactions were incubated at 37 °C for 1 h and subsequently incubated at 80 °C for 10 min to deactivate the DNA polymerase. After the extension step, the reaction mixture was divided into two portions. The first 10 uL of extension reaction was used as the cleavage reaction and was added with 5 U of Nb.Bpu10I nicking endonuclease and 10 uL of 1× Buffer R. The remaining 10 uL was added with 10 uL of 1× buffer R and used as the negative control. The cleavage and control reactions were incubated at 37 °C for 3 h. Bsm DNA polymerase, Buffer R, and Nb.Bpu10I nicking endonuclease were purchased from Thermo Fisher Scientific Inc. (Waltham, MA, USA).

### Exponential isothermal amplification

Two reaction mixtures were prepared separately on ice. The first mixture contained RNA sample, external control miRNA (mir-159a), Buffer R, dNTPs, stem-loop probes, and amplifiers. The second mixture contained Bsm DNA polymerase and Nb.Bpu10I nicking endonuclease. The two mixtures were combined at 37 °C and incubated for 3 h. The final reaction mixture contained 20 μL of varying amounts of synthetic miRNA or 800 ng of total RNA, 20 fmol of external control miRNA, 60 fmol of stem loop probes, 30 fmol of amplifier, 0.5 U of Bsm DNA polymerase, 7 U of Nb.Bpu10I, 1× Buffer R, and 250 μM dNTPs. Subsequent linear amplification was performed in 20-μL reaction volumes containing PCR Premix kit (Bioneer, Daejeon, Korea), 2 μL of miRNA assay reagent, and 20 fmol of primer (5′-FAM-GTGCCAGCAAGATCCAATCTAGA-3′) to label the amplified products. Reactions were carried out with the following amplification profile: initial denaturation at 95 °C for 5 min; 20 cycles of 95 °C for 30 s, 60 °C for 30 s, and 72 °C for 40 s; and final extension at 72 °C for 7 min.

### CE and CE-SSCP

Denaturing DNA fragment analysis was performed on an ABI 3130xl Genetic analyzer (36 cm capillary array and POP4 polymer; Applied Biosystems Inc., Foster City, CA, USA). Sample mixtures contained 1 μL of assay product, 0.2 μL of ROX 500 size-standard (Applied Biosystems, Inc.), and 8.8 μL of deionized formamide (Applied Biosystems). Samples were denatured at 95 °C for 5 min and quenched at 4 °C for 3 min. Mixtures were then injected into capillaries by applying an injection voltage of 1.6 KV for 15 s, and samples were resolved at an electrophoresis voltage of 10 kV and operating temperature of 60 °C.

For CE-SSCP analysis, which was performed in non-denaturing conditions, samples were prepared by mixing 1 μL of 100-fold diluted assay product with 0.2 μL of ROX 500 size-standard and 8.8 μL of deionized formamide. Resulting mixtures were denatured at 95 °C for 5 min and quenched at 4 °C for 3 min. Non-denaturing electrophoresis was performed on an ABI 3130xl Genetic Analyzer (50-cm capillary array and 15% Pluronic F108 polymer solution) The Pluronic F108 polymer (Product no. 542342; Sigma-Aldrich, St. Louis, MO, USA) solution was prepared as described in our previous report^22^. Briefly, the Pluronic F108 polymer was dissolved in 0.7 × 310 Running Buffer at 15 wt%. Then, the polymer solution was centrifuged at 3500 rpm for 30 min at 4 °C to remove the bubbles. Sample mixtures were injected into capillaries by applying an injection voltage of 15 kV for 5 sec and resolved at an electrophoresis voltage of 15 kV and an operating temperature of 35 °C.

### Data analysis

The target peak was identified based on electrophoretic mobility obtained by GeneMapper v4.1 (Applied Biosystems), with which we calibrated the mobility based on the size standards mixed in every electrophoresis run. All the target peaks have highly reproducible mobility after the calibration (Supplementary Table S3). miRNA expression levels were determined by dividing the area of each target peak by the total peak area of reference probes (mir-159a and snoRNA U18). mir-159a was used as spike-in control, and snoRNA U18 was used as an endogenous control. The individual peak area for each small RNA were obtained by Gene Mapper v4.1 software (Applied Biosystems). Karp *et al*. also used snoRNA U18 as an internal control for qRT-PCR assays, and their findings were comparable with our assay results^8^. All miRNA assays were performed with at least three replicates.

## Electronic supplementary material

Supplementary Information
