# Supplementary material for: Multiplex quantitative analysis of microRNA expression via exponential isothermal amplification and conformation-sensitive DNA separation
Source: Sci Rep. 2017 Sep 12;7:11396. doi: 10.1038/s41598-017-11895-6 (PMC5595994; doi:10.1038/s41598-017-11895-6)

**Supplementary Information**

**Multiplex quantitative analysis of microRNA expression via exponential isothermal amplification and conformation-sensitive DNA separation**

Jeongkyeong Na^1^, Gi Won Shin^2‡^, Heehwa G. Son^3^, Seung-Jae V. Lee^3,4, 5,*^ and Gyoo Yeol Jung^1,5,*^

^1^ Department of Chemical Engineering, Pohang, Gyeongbuk, 790-784, Korea. ^2^ Institute of Environmental and Energy Technology, Pohang University of Sciences and Technology, Pohang, Gyeongbuk, 790-784, Korea. ^3^ Department of Life Sciences, Pohang University of Sciences and Technology, Pohang, Gyeongbuk, 790-784, Korea. ^4^ Information Technology Convergence Engineering, Pohang University of Science and Technology, Pohang, Gyeongbuk, 790-784, Korea. ^5^ School of Interdisciplinary Bioscience and Bioengineering, Pohang University of Sciences and Technology, Pohang, Gyeongbuk, 790-784, Korea

*To whom correspondence should be addressed: Gyoo Yeol Jung; Tel: +82 54 279 2931; Fax: +82 54 279 5528; Email: gyjung@postech.ac.kr, Seung-Jae V. Lee. Tel: +82 54 279 8066; Email: seungjaelee@postech.ac.kr

‡ Current affiliation: Division of Oncology, Department of Medicine, Stanford University School of Medicine, Stanford, California 94305, USA

|  | Label | Sequence | Length (bp) |
| --- | --- | --- | --- |
| miRNAs involved in  developmental timing | lin-4 | UCCCUGAGACCUCAAGUGUGA | 21 |
|  | mir-48 | UGAGGUAGGCUCAGUAGAUGCGA | 23 |
|  | mir-84 | UGAGGUAGUAUGUAAUAUUGUAGA | 24 |
| Tissue-specific miRNAs | lsy-6 | UUUUGUAUGAGACGCAUUUCG | 21 |
|  | mir-55 | UACCCGUAUAAGUUUCUGCUGAG | 23 |
|  | mir-56 | UACCCGUAAUGUUUCCGCUGAG | 22 |
| Internal controls | U18 | UGGCAGUGAUGAUCACAAAUCCGUGUUUCUGACAAGCGAUUGACGAUAGAAAACCGGCUGAGCCA | 65 |
|  | mir-159a | UUUGGAUUGAAGGGAGCUCUA | 21 |

Table S1. microRNA sequences used for analysis.

**Table S2. Stem-loop probes and amplifiers used for multiplex microRNA analysis.**

1. **Stem-loop probes**

|  | Label | 5'- Trigger | Stem-loop sequences | Nicking site | Anti-miRNA -3' | Length |
| --- | --- | --- | --- | --- | --- | --- |
| miRNAs involved in developmental timing | lin-4 | GCTGATGCTATG | GTCGTATCCAGTGCAGGGTCCGAGGTATTCGCACTGGATACGAC | CCTCAGC | TCACACTTGAGGTCTC | 79 |
|  | mir-48 | CGTGAGTGATGA | GTCGTATCCAGTGCAGGGTCCGAGGTATTCGCACTGGATACGAC | CCTCAGC | TCGCATCTACTGAGCC | 79 |
|  | mir-84 | CCATCCACACA | GTCGTATCCAGTGCAGGGTCCGAGGTATTCGCACTGGATACGAC | CCTCAGC | TCTACAATATTACATACTACC | 83 |
| Tissue-specific miRNAs | lsy-6 | CGGATCGTAAA | GTCGTATCCAGTGCAGGGTCCGAGGTATTCGCACTGGATACGAC | CCTCAGC | CGAAATGCGTCTCATAC | 79 |
|  | mir-55 | GCCTTCAACG | GTCGTATCCAGTGCAGGGTCCGAGGTATTCGCACTGGATACGAC | CCTCAGC | CTCAGCAGAAACTTA | 76 |
|  | mir-56 | CCAGTCTGCG | GTCGTATCCAGTGCAGGGTCCGAGGTATTCGCACTGGATACGAC | CCTCAGC | CTCAGCGGAAACATTAC | 78 |
| Internal controls | U18 | CCTGCAGTACC | GTCGTATCCAGTGCAGGGTCCGAGGTATTCGCACTGGATACGAC | CCTCAGC | TGGCTCAGCCGGTTT | 77 |
|  | mir-159a | CGCCAGATTC | GTCGTATCCAGTGCAGGGTCCGAGGTATTCGCACTGGATACGAC | CCTCAGC | TAGAGCTCCCTTCAATC | 78 |

1. Amplifiers

|  | Label | 5'- Anti-miRNA | Common primer | Variable sequences | Nicking site | Trigger -3' | Length |
| --- | --- | --- | --- | --- | --- | --- | --- |
| miRNAs involved in developmental timing | lin-4 | TCACACTTGAGGTCTC | GTGCCAGCAAGATCCAATCTAGA | CCTCTGCGCTCTACATACAACATCTCTTTC | CCTCAGC | GCTGATGCTATG | 88 |
|  | mir-48 | TCGCATCTACTGAGCC | GTGCCAGCAAGATCCAATCTAGA | CCATGAATCCTTGAATTGTTCCATCTTCACC | CCTCAGC | CGTGAGTGATGA | 89 |
|  | mir-84 | TCTACAATATTACATACTACC | GTGCCAGCAAGATCCAATCTAGA | TTTAGCATATAGACGATTCTCCCCTCTGCGCTC | CCTCAGC | CCATCCACACA | 95 |
| Tissue-specific miRNAs | lsy-6 | CGAAATGCGTCTCATAC | GTGCCAGCAAGATCCAATCTAGA | AGCTCAAGAGTTGCCCATCCTGCAGCAATGTTATTCCCTG | CCTCAGC | CGGATCGTAAA | 98 |
|  | mir-55 | CTCAGCAGAAACTTA | GTGCCAGCAAGATCCAATCTAGA | CATCGTCTTTATCTCCGAGCTCAGAAATTATTCTCGCCTG | CCTCAGC | GCCTTCAACG | 95 |
|  | mir-56 | CTCAGCGGAAACATTAC | GTGCCAGCAAGATCCAATCTAGA | TCTTGCATGCCTTTAGCCATTGCTTCCGGACTAACATC | CCTCAGC | CCAGTCTGCG | 95 |
| Internal controls | U18 | TGGCTCAGCCGGTTT | GTGCCAGCAAGATCCAATCTAGA | TCACTGATTGCGCCGAAGTTTACATTCAAGC | CCTCAGC | CCTGCAGTACC | 87 |
|  | mir-159a | TAGAGCTCCCTTCAATC | GTGCCAGCAAGATCCAATCTAGA | AGCAATTGCCGCTTCAATTTCACCAAGCTCAATTCGGAAG | CCTCAGC | CGCCAGATTC | 97 |

**Table S3.** Reproducibility of eight small RNAs’ peak position.

|  | Migration time (AU)* | | CV**  (STD/Avg) |
| --- | --- | --- | --- |
|  | Average | STD |  |
| lin-4 | 7389.87 | 119.31 | 1.61% |
| mir-48 | 7446.13 | 116.66 | 1.57% |
| mir-84 | 7517.09 | 125.99 | 1.68% |
| lsy-6 | 7810.17 | 154.98 | 1.98% |
| U18 | 7674.87 | 75.75 | 0.99% |
| mir-159a | 7941.59 | 145.90 | 1.84% |
| mir-55 | 7978.95 | 129.56 | 1.62% |
| mir-56 | 8208.08 | 138.33 | 1.69% |

*Migration time is calibrated with GeneMapper v4.1 (Applied Bioscience) and represents relative position of small RNA’s peak

**CV (Coefficient of variation): Standard deviation divided by average

**Supplementary Figure S1**. Detailed schemes for Cycles 1, 2, and 3 during isothermal exponential amplification (EXPAR) of miRNAs. **(a)** Designs for the stem-loop probe and amplifier used in the miRNA assay. The stem-loop probes, which are used to generate triggers in Cycles 1 and 3, consist of anti-miRNA, nicking site, and trigger sequences. Amplifiers are used to generate signal barcodes, which are separated and detected via conformation-sensitive capillary electrophoresis (CE). To detect signal barcodes in CE, the products must be labeled with a fluorescent tag. Variable sequences were also designed for CE-SSCP analysis. CE-SSCP separates multiple signal barcodes based on single-strand DNA conformation in non-denaturing conditions. Therefore, the amplifier is designed to contain a partial trigger, nicking site, variable sequences, common primer, and anti-miRNA. **(b)** Steps in Cycle 1 for generating triggers. When a miRNA hybridizes to the stem-loop probe, the miRNA is extended using the probe as template. The newly synthesized strand is cleaved by Nb.Bpu 10I at the nicking site; the cleaved fragment, the trigger, is released via strand displacement by Bsm DNA polymerase. Through repetitive extension, cleavage, and single-strand displacement steps, triggers are amplified in proportion to the starting amounts of the miRNAs. **(c)** Steps in Cycle 2 for generating signal barcodes. When the trigger generated in Cycle 1 hybridizes to the amplifier, signal barcodes are amplified in proportion to the amount of trigger through the three repetitive steps. **(d)** Steps in Cycle 3 for generating additional triggers. This cycle completes exponential amplification of signal barcodes. When the signal barcode generated in Cycle 2 hybridizes to stem-loop probe, the additional triggers are generated.

**Supplementary Figure S2.** Denaturing CE analysis (size-based DNA separation) of extended and singly nicked products in Cycles 1, 2, and 3 of isothermal EXPAR. Expected extended and cleaved products for 7 small RNAs (lin-4, mir-84, snoRNA U18, lsy-6, mir-159a, mir-55, and mir-56) in each cycle were confirmed via denaturing CE analysis. Extended products were confirmed by adding Bsm DNA polymerase without Nb.Bpu10I. Extended and singly nicked products were generated by adding Nb.Bpu10I to the extension reaction. The expected products are indicated by black arrows in electropherograms.

**Supplementary Figure S3**. Determination of the optimal combination of enzyme quantities in the miRNA assay. The signal intensity representing the yield of isothermal EXPAR is plotted for five different Nb.Bpu10I amounts. Two different Bsm DNA polymerase amounts were also tested. The amplification efficiency increased with increasing amount of nicking enzyme for up to 7 U. Optimal signal intensity was obtained with 0.5 U of Bsm DNA polymerase and 7 U of Nb.Bpu10I.

**Supplementary Figure S4**. Validity of multiplex miRNA assay. Various amount of individual target miRNAs are mixed with a standard miRNA (mir-159a), and analyzed by the multiplex miRNA assay. X- and Y-axes correspond to migration time in arbitrary units (AUs) and relative fluorescence units (RFU), respectively.

**Supplementary Figure S5.** Correlation between the expected miRNA fold changes and of signal intensity ratio. Various fold changes were simulated by mixing seven synthetic miRNAs. Twofold, fivefold, and tenfold increases in the expression of all six target miRNAs were quantified using our multiplex miRNA assay. Mean and standard deviation of triplicate measurements are plotted. Expected fold changes and signal intensity ratios are indicated in the x-axis and y-axis, respectively.


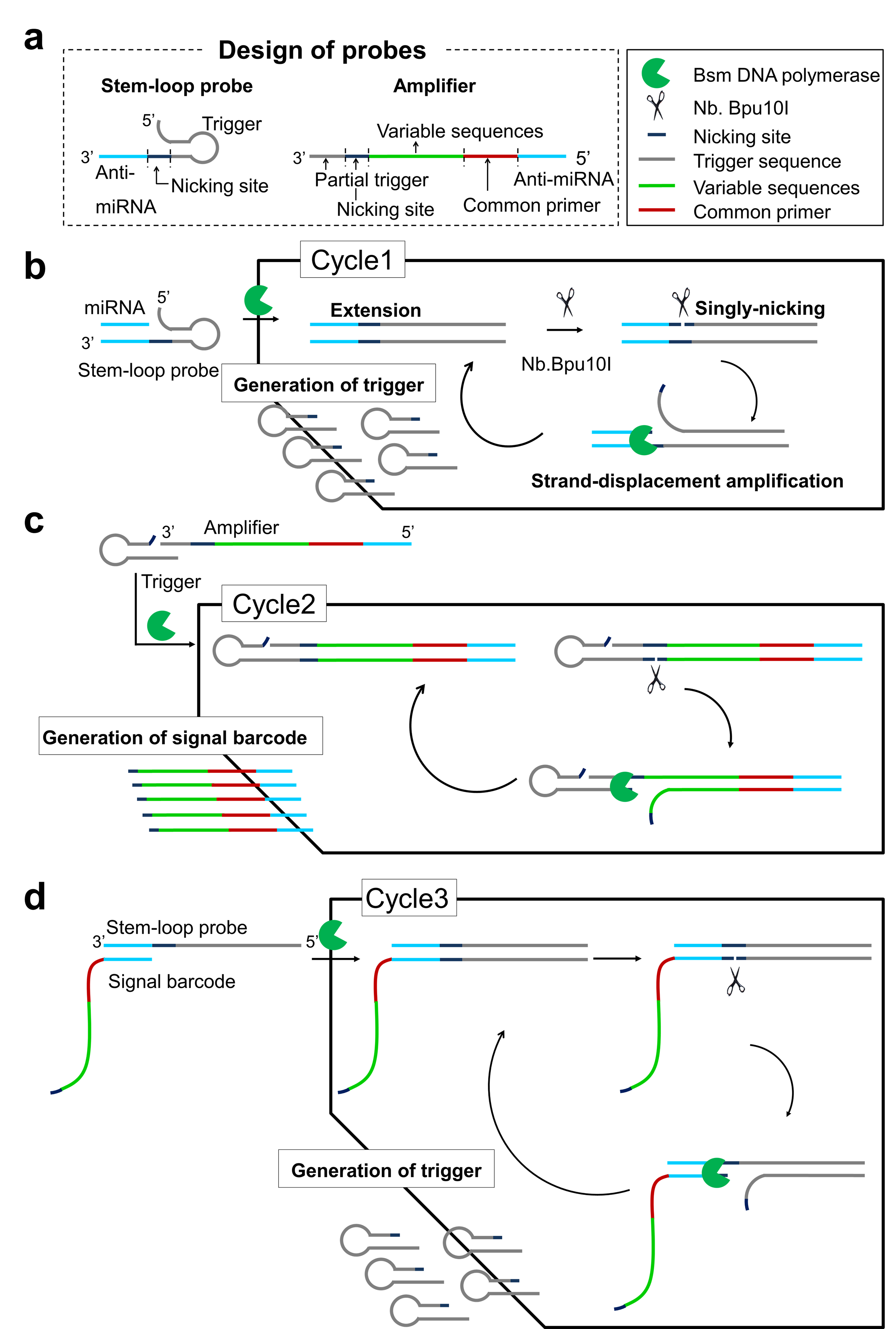


Supplementary Figure S1


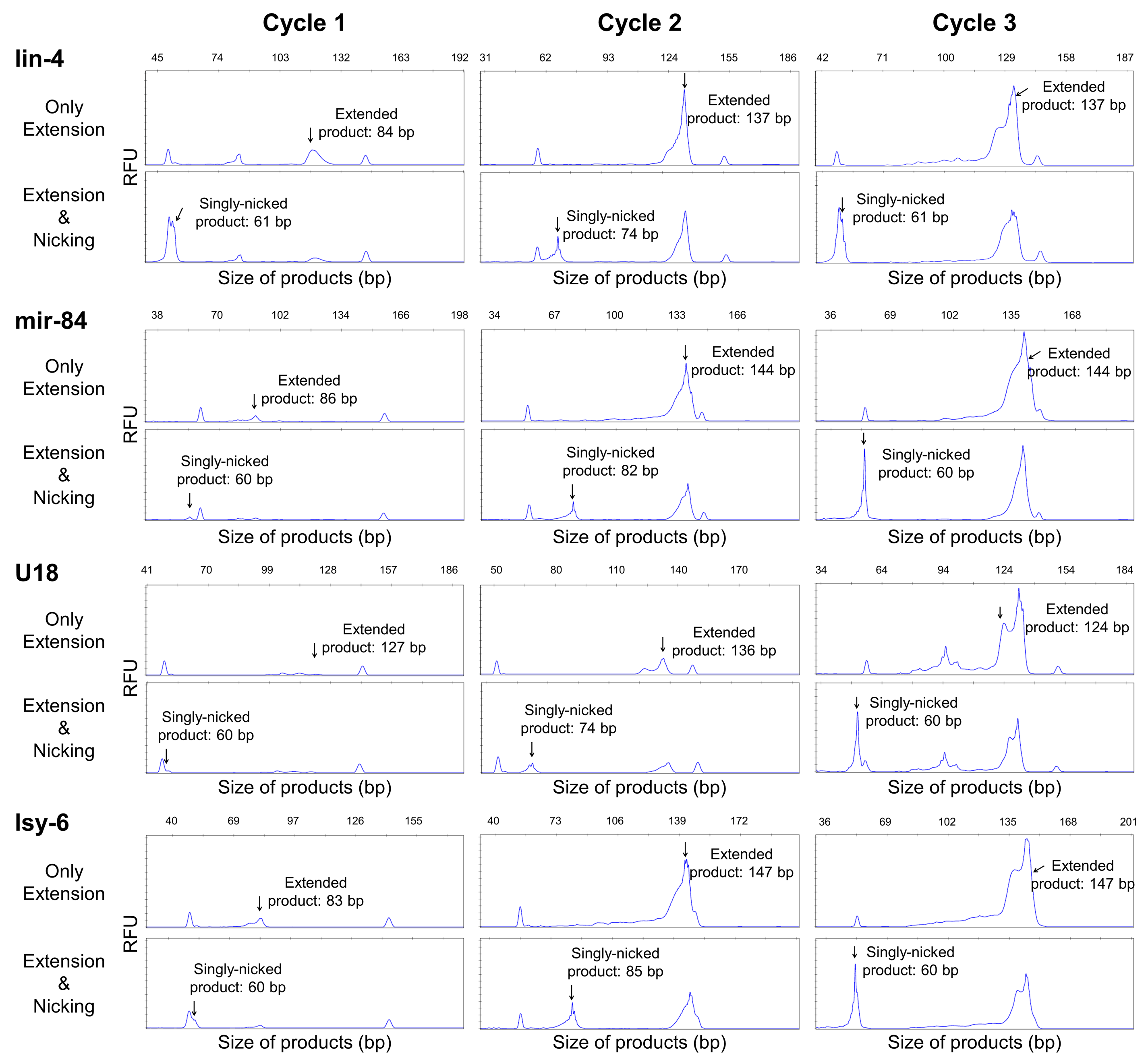


Supplementary Figure S2


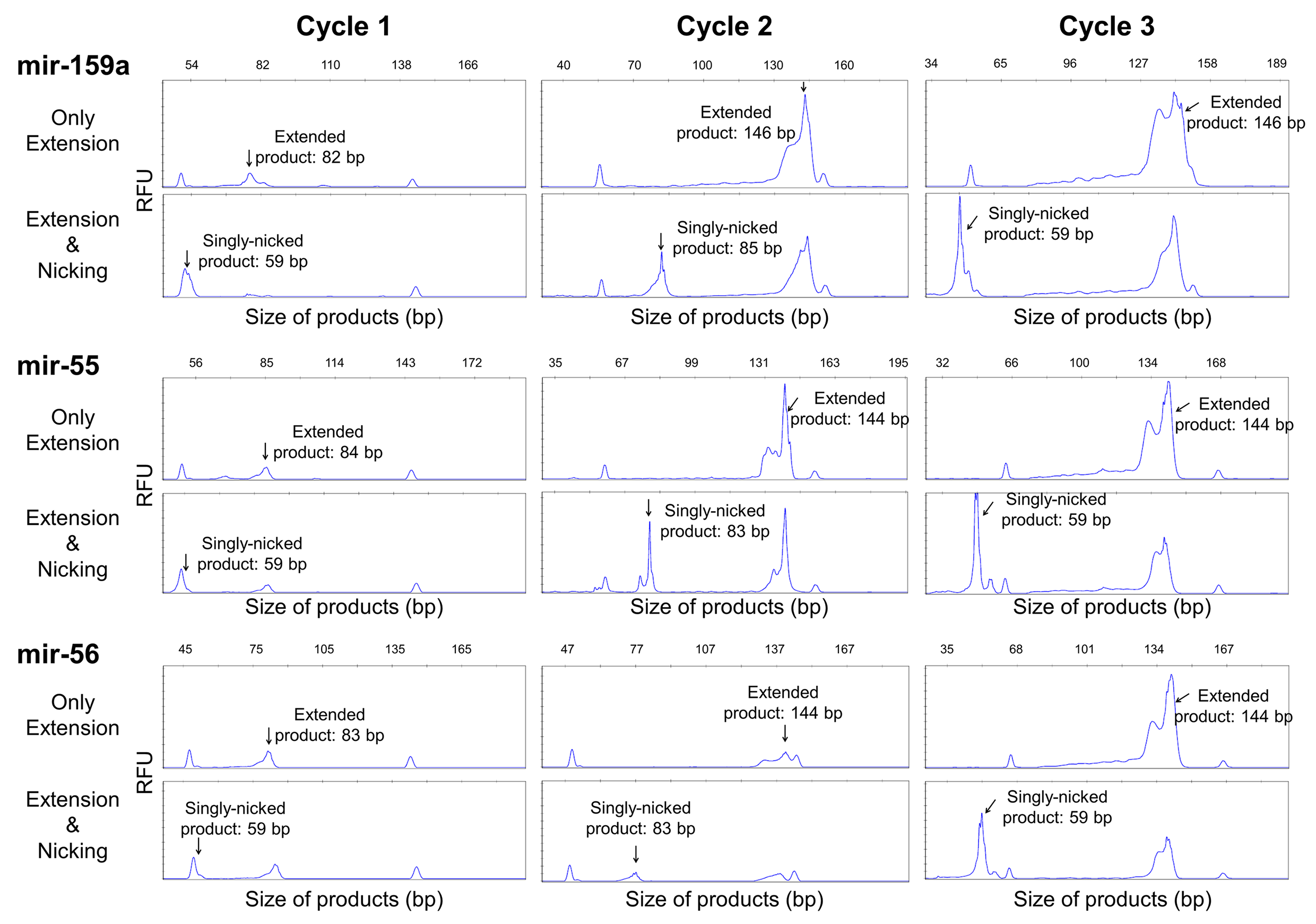


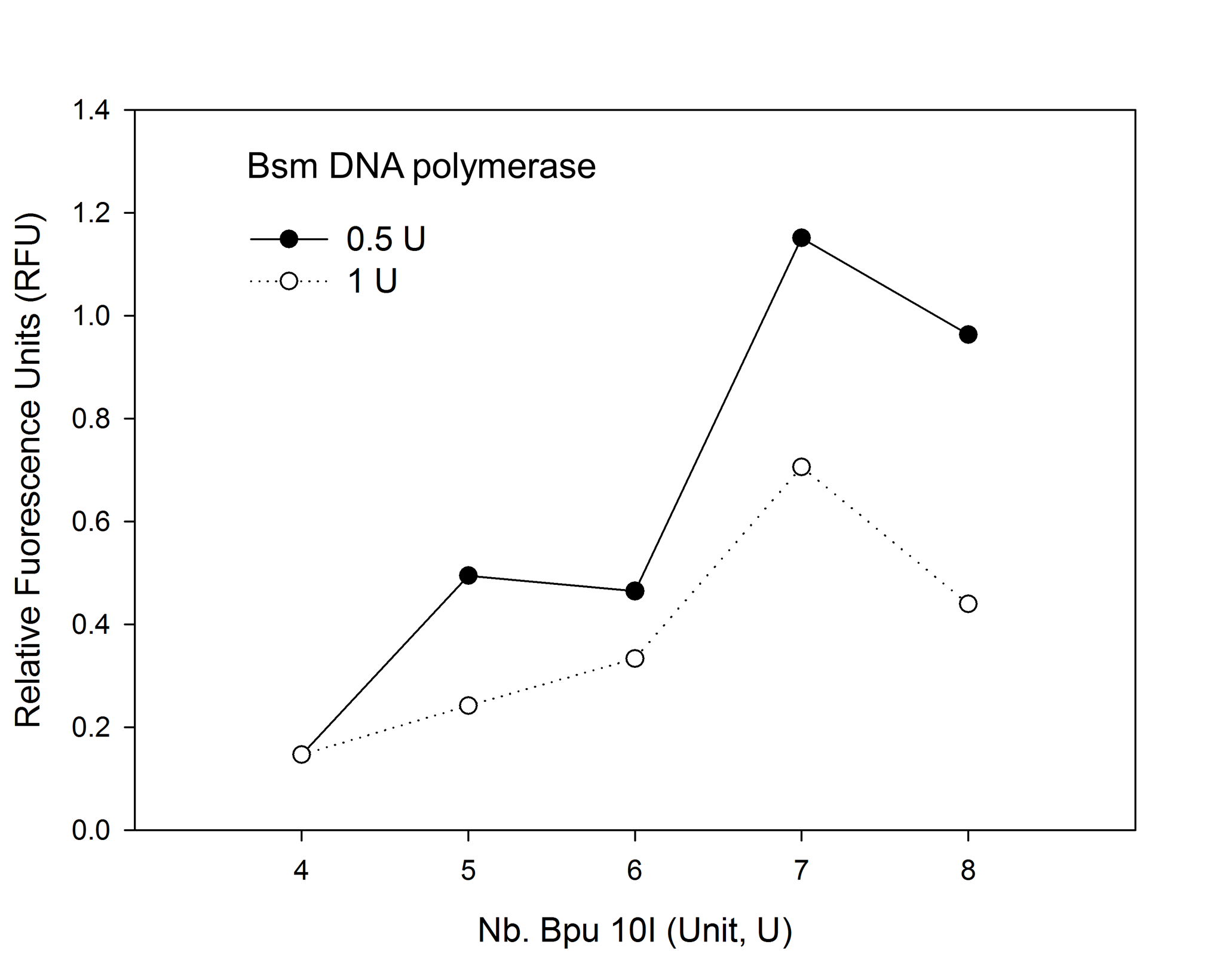


Supplementary Figure S3


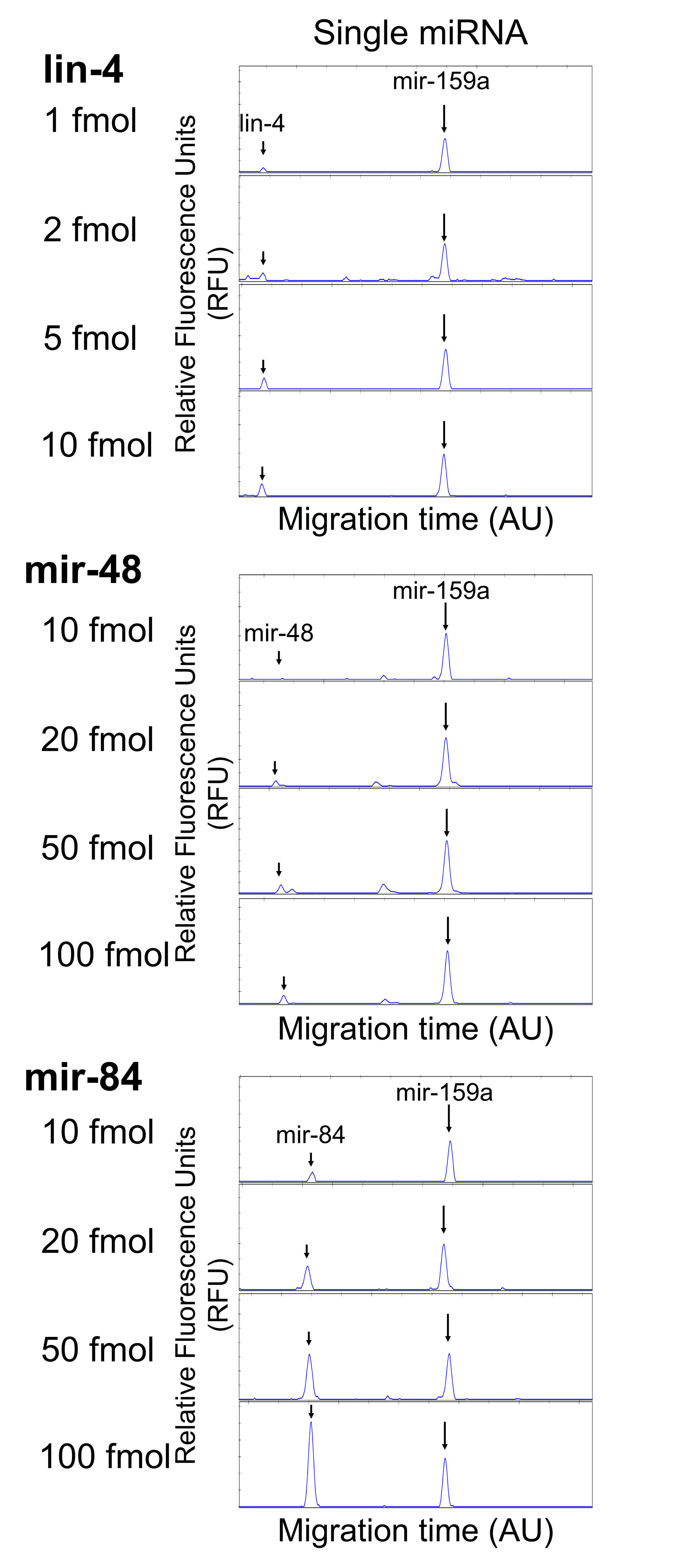


Supplementary Figure S4


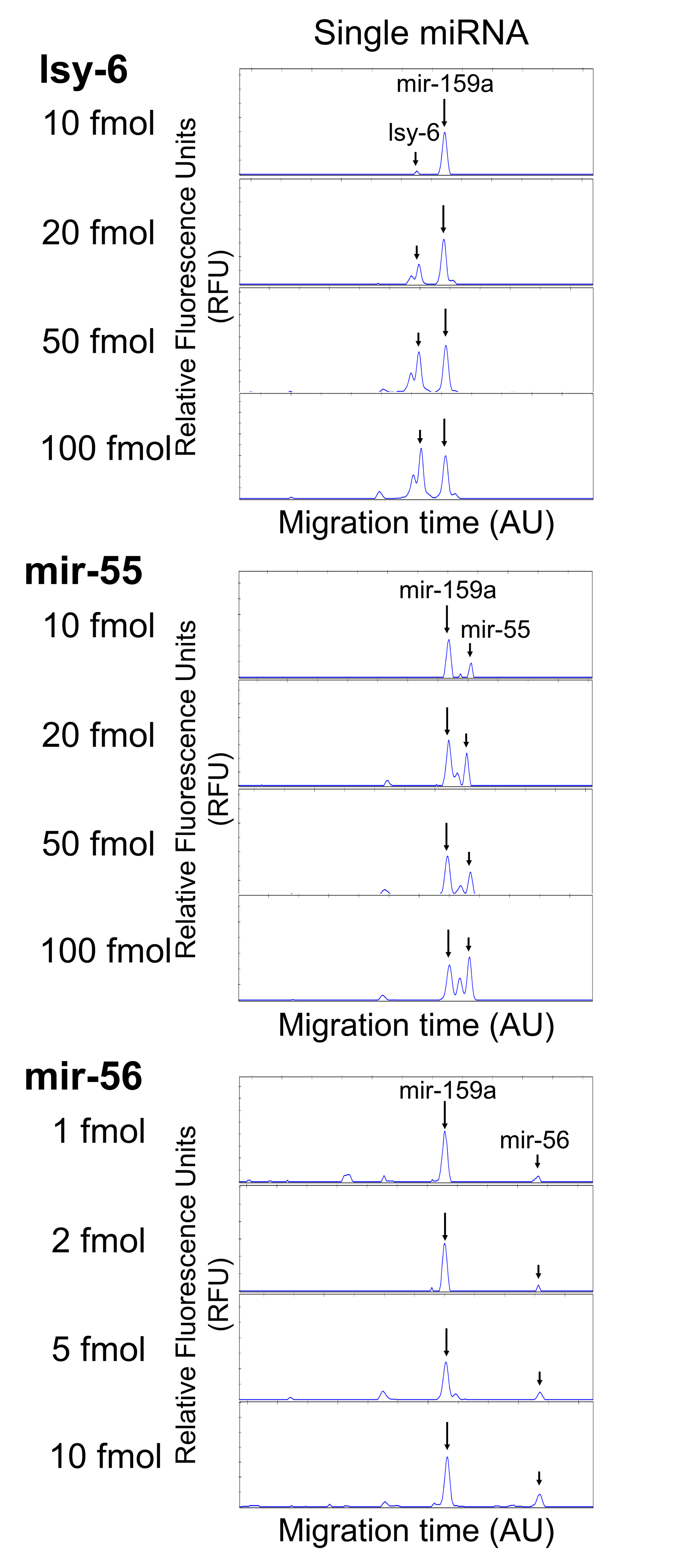


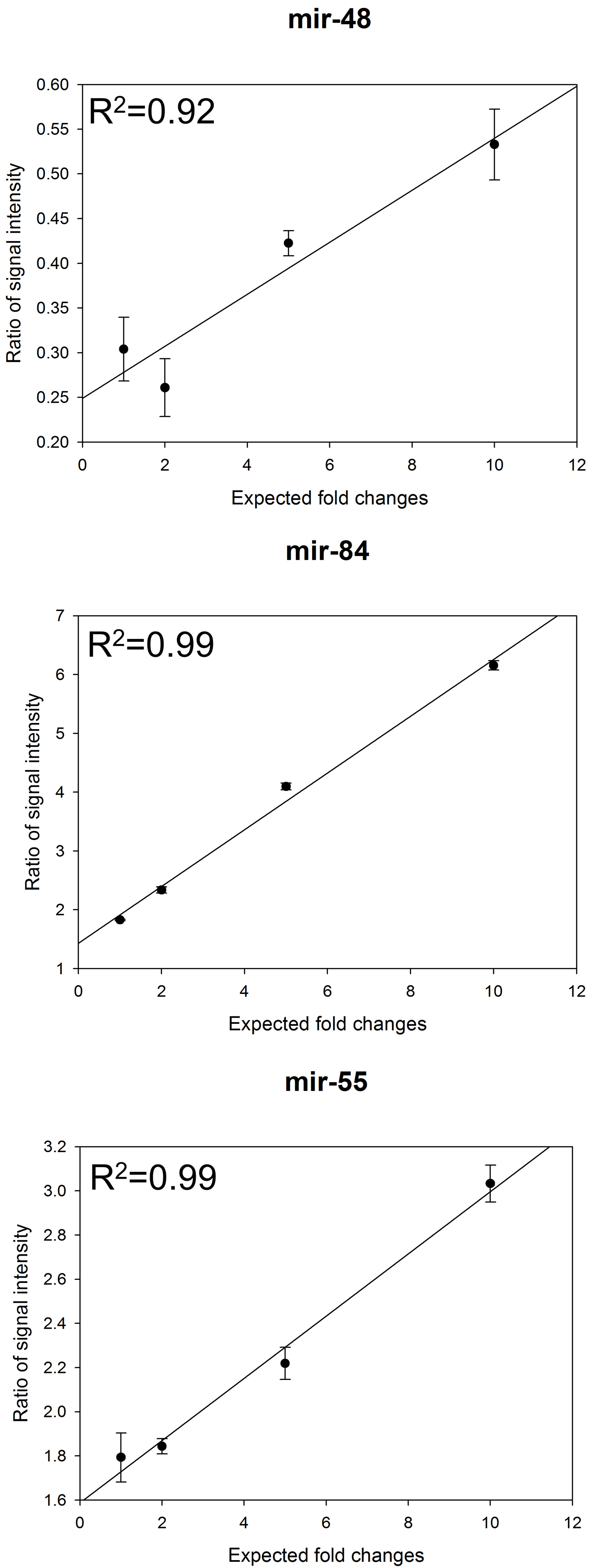


Supplementary Figure S5


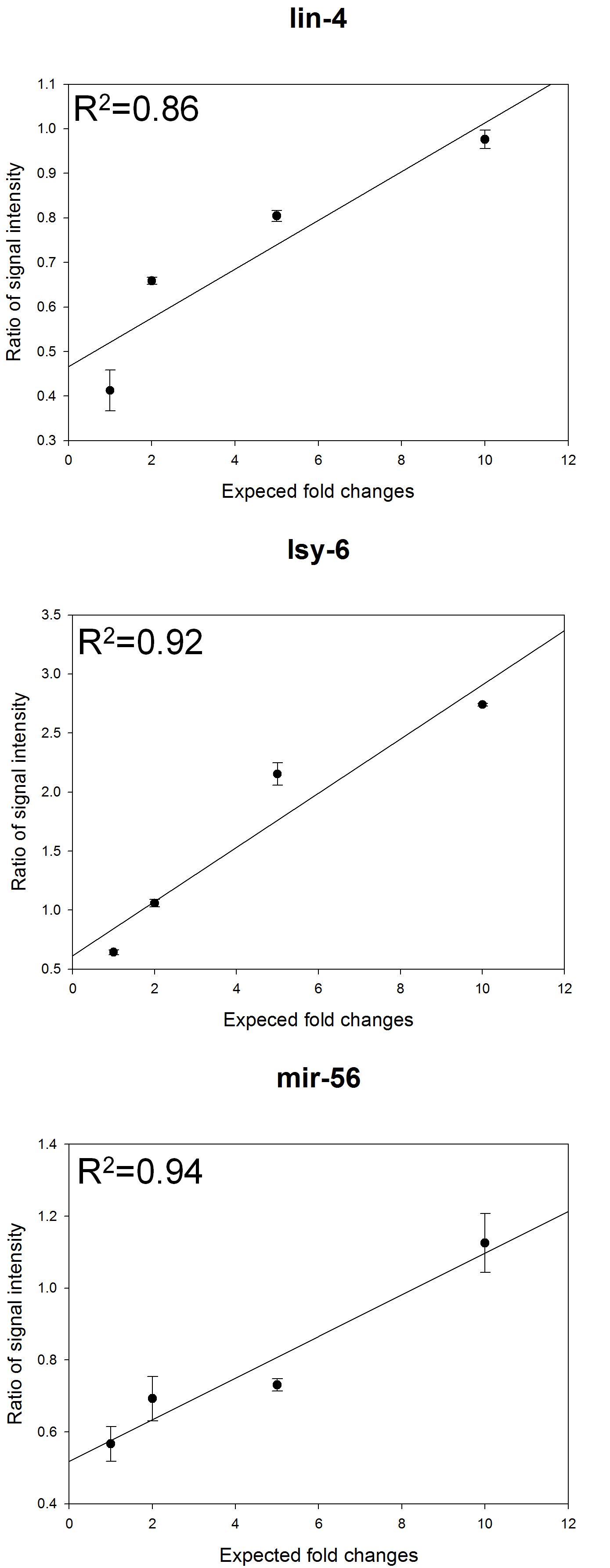

Supplement: Supplementary file 1 — Supplementary Information [file 41598_2017_11895_MOESM1_ESM.doc]
